# *In-vitro* Modulation of mTOR-HIF-1α Axis by TLR7/8 Agonist (Resiquimod) in B-Chronic Lymphocytic Leukemia

**DOI:** 10.1007/s12288-023-01649-y

**Published:** 2023-04-07

**Authors:** Rana M. Hanafy, Soheir R. Demian, Lobna A. Abou-Shamaa, O. Ghallab, Eman M. Osman

**Affiliations:** 1 Immunology and Allergy Department, Medical Research Institute, Alexandria University, Alexandria, Egypt; 2 Internal Medicine Department (Hematology Unit), Faculty of Medicine, Alexandria University, Alexandria, Egypt

**Keywords:** B cells, Immunometabolism, mTOR, HIF-1α, B-CLL, TLR7/8 agonist

## Abstract

Targeting toll-like receptors (TLRs), via TLR agonists, has been implicated in the regulation of immunometabolism. B-chronic lymphocytic leukemia (B-CLL) represents a suitable model for B-cell derived malignancies with shifted metabolic adaptations. Several signaling pathways have been found to be critical in metabolic reprogramming of CLL, including mechanistic target of rapamycin- hypoxia inducible factor-1α (mTOR- HIF-1α) pathway, the main metabolic regulator of glycolysis. Here, we investigated the effect of TLR7/8 agonist (Resiquimod) on the expression of mTOR and HIF-1α in patients with CLL. B cells were purified using Rosettesep Human B cell Enrichment Cocktail (Stem cell Technologies, Vancouver, BC, Canada#15,024) from peripheral venous blood of CLL patients (n = 20) and healthy individuals (n = 15). Isolated B cells were then cultured in both presence and absence of Resiquimod. Gene expression of mTOR and HIF-1α were assessed using qRT-PCR. Resiquimod significantly decreased mTOR and HIF-1α gene expression in both CLL (*p* < 0.001and *p* < 0.001, respectively) and Normal B cells (*p* = 0.004 and *p* = 0.001, respectively). Resiquimod may reprogram immunometabolism of malignant B-CLL cells via down-regulation of key glycolytic metabolic actors, mTOR and HIF-1α genes. Accordingly, Resiquimod may be an adjuvant as a therapeutic tool for CLL, which needs to be studied further.

**Supplementary Information:**

The online version contains supplementary material available at 10.1007/s12288-023-01649-y.

## Introduction

B-chronic lymphocytic leukemia (CLL) is the most common type of leukemia in adults [1]. It is characterized by clonal proliferation and accumulation of malignant B-lymphocytes in the peripheral blood and in immune tissues such as lymphoid organs and bone marrow [2]. It has been reported that B cells, isolated from peripheral blood of CLL patients, express high and intermediate levels of TLR7 and TLR8, respectively [3]. Being highly malignant, CLL cells are characterized by shifted immunometabolic adaptations supporting malignant cell proliferation and resistance to apoptosis. Several signaling pathways have been found to be critical in metabolic reprogramming of CLL, including mTOR-HIF-1α pathway [4]. Thus, development of novel immunotherapeutic agents that interfere with any of these signaling pathways would be of great interest.

Altered metabolism plays an important role in the malignant biological behaviors of different types of cancer. This contributes in favoring the survival, proliferation, invasion, metastasis and chemoresistance of cancer cells. For instance, tumor cells use glycolysis, even in the presence of oxygen and fully functioning mitochondria. This process of aerobic glycolysis or “*Warburg effect*” has been suggested to be an adaptation mechanism supplying tumor cells with their biosynthetic requirements [5]. Mechanistic target of rapamycin (mTOR) is a key metabolic regulator of multiple cellular functions including cell proliferation, differentiation, nutrient uptake and energy metabolism. In response to fluctuating levels of nutrients and energy, mTOR regulates cell growth through coordination of different metabolic pathways such as lipid and protein synthesis [6]. At the molecular level, mTOR directly stimulates mRNA translation and ribosomal bio-macromolecular synthesis of other key metabolic transcription factors such as hypoxia inducible factor-1 alpha (HIF-1α), a master regulator of oxygen homeostasis [7].

Toll-like receptors (TLRs) are prototypic pattern recognition receptors (PRRs) [8]. The latter are responsible for induction of specific immune response to pathogen associated molecular patterns (PAMPs) and damage associated molecular patterns (DAMPs) [9]. Till now, ten functional TLRs subtypes (i.e. TLR1-TLR10) have been found to be expressed in humans that can be further functionally categorized according to subcellular localization and sensed ligands. TLR1, TLR2, TLR4, TLR5, TLR6 and TLR10 comprise the group localized at the cell surface. Alternatively, TLR3, TLR7, TLR8 and TLR9 are expressed intracellularly, particularly in endosomal microcompatment [8]. It has been proposed that signaling via TLRs is a critical factor involved in carcinogenesis, regulating both tumor cells and tumor-infiltrating immune cells [10]. Of note, the majority of clinical trials, phases (1, 2 and 3), have evaluated TLR ligands as immune adjuvants to improve the immunogenicity of various cancer or immunomodulatory vaccines; such as TLR3 agonist in low grade B-cell lymphoma, melanoma and metastatic colon cancer and TLR7 agonist in Hepatitis B vaccine (HBV) and Human papilloma virus (HPV) vaccine[11]. Resiquimod is a TLR7/8 agonist mainly applied as an adjuvant with immunomodulatory effect in the context of cancer therapy (e.g. metastatic lung cancer, colon cancer) [12]. This is done via induction of interferon α (IFNα), interleuk-12 (IL-12) and tumor necrosis factor-α (TNF-α) [13]. In addition, it results in stimulation of antigen-specific- cell mediated immune response via activation of natural killer (NK) cells with indirect induction of IFN-γ [14].

Although previous studies investigated the effect of multiple TLRs on CLL cells, targeting the metabolic pathways employed by malignant and normal B cells using TLRs, particularly TLR7/8 agonist, represents a novel research point. In our study, we examined the effect of Resiquimod on metabolic mediators (mTOR and HIF-1α) in normal B cells isolated from healthy individuals and malignant B cells isolated from CLL patients.

## Materials & Methods

### Study Population

The current study was conducted on twenty CLL patients (CLL group) and fifteen healthy normal age and sex matched individuals (Normal group) (Table 1). All individuals under study were subjected to complete history taking and thorough clinical examination. All participants were asked to freely volunteer in the study and informed written consents were gathered prior to their inclusion in study protocol according to ESM (Supplementary material).

**Table 1:** Demographic data of study population

	CLL (n = 20)	Normal (n = 15)	*P*
Sex (n)			0.181
Male	15	8	
Female	5	7	
Age (year)			0.692
Min–Max	40.0–67.0	51.0–69.0	
Mean ± SD	58.40 ± 6.52	57.60 ± 4.82	

B-CLL were diagnosed according to the standard clinical and laboratory criteria [15]. Patients were either untreated or did not receive treatment for three months before enrolment in the study and any other malignancy or autoimmune diseases were excluded. CLL patients were classified into three groups according to Rai staging system as shown in (Table 2).

**Table 2:** Distribution of CLL patients (n = 20) according to Rai Staging

Rai stages	No.	%
Low risk	1	5.0
Intermediate risk	8	40.0
High risk	11	55.0

### Peripheral Blood Samples

Peripheral venous blood samples were collected from fasting patients (1 ml) or normal individuals (20 ml, to overcome the problem of relative lower B cell count) into sterile heparinized vacutainers. For normal samples, peripheral blood mononuclear cells (PBMCs) were first isolated using centrifugation over ficoll-hypaque density medium and then re-suspended in 1 ml autologous whole blood for further B cell isolation [16].

### Isolation of Peripheral B Cells

B cells were isolated by negative selection using RosetteSep Human B cell Enrichment Cocktail (Stem cell Technologies, Vancouver, BC, Canada#15,024) following the manufacturer’s instructions. In short, The RosetteSep™ antibody cocktail crosslinks unwanted non-B cells in human whole blood to multiple RBCs, forming immunorosettes. This increases the density of the unwanted (rosetted) cells, such that they pellet along with the free RBCs when centrifuged over ficoll-hypaque density medium. Desired cells are never labeled with antibody and are easily collected as a highly enriched population at the interface between the plasma and the density gradient medium [17]. Cell viability and numbers were then determined using Trypan Blue dye exclusion technique.

### In vitro Treatment of B cells Resiquimod

B cells were treated with Resiquimod according to [18] with minor modifications. Briefly, isolated B cells (2 × 10^6 ^cells/ml/well) were suspended in complete culture media (RPMI supplemented with 10% heat inactivated fetal bovine serum and (1%) penicillin/streptomycin) and cultured in both presence and absence of 1 μg/ml Resiquimod (Sigma, USA). Cultured cells were incubated for 48 h at 37 °C in a humidified 5% CO_2 _incubator. At the end of the culture period, culture yield was collected and stored at − 80 °C for RNA extraction and subsequent gene expression determination.

### Gene Expression mTOR and HIF-1α mRNA in B Cells

First, total cellular RNA was extracted using QIAamp RNA Mini Kit (Qiagen, Germany) according to manufacturer recommendation. Total extracted RNA concentration and purity was determined using Nano Drop Thermo spectrophotometry (Thermo Scientific, USA). Second, Reverse transcription was done using RevertAid First Strand cDNA Synthesis Kit (Thermo scientific, USA) according to manufacturer recommendations. Finally, Quantitative-real time-PCR for quantification of mTOR [19] and HIF-1α [20] gene expressions were performed using StepOne™ Real Time PCR system, (Applied Biosystems, Foster city, USA).

Differences in threshold cycle (Ct) values between gene of interest and housekeeping gene GAPDH (∆Ct) were converted into fold gene expression by 2^−∆∆Ct^ using the following equations:

$$\begin{gathered} {\text{Delta Ct }}\left( {\Delta {\text{Ct}}} \right) \, = {\text{ Ct}}_{{({\text{target}}\;{\text{ gene}})}} {-}{\text{ Ct}}_{{\left( {{\text{housekeeping}}\;{\text{ gene}}} \right)}} \hfill \\ {\text{Delta Delta Ct }}\left( {\Delta \Delta {\text{Ct}}} \right) \, = \, \Delta {\text{Ct}}_{{({\text{target gene}})}} - \, \Delta {\text{Ct}}\;{\text{ of }}\;{\text{calibrator}}_{{\left( {{\text{average}}\; \, \Delta {\text{Ct }}\;{\text{of}}\;{\text{ untreated}}\;{\text{ control}}} \right)}} \hfill \\ \end{gathered}$$

The expression of both genes in Normal group in absence of TLR7/8 agonist was considered as 1 (normalization). The corresponding values of gene expression in presence and absence of TLR7/8 agonist in (Normal/CLL groups) and (CLL group), respectively were calculated as a fold change [21].

### Statistical Analysis

Data were fed to the computer and analyzed using IBM SPSS software package version 20.0*.* (Armonk, NY: IBM Corp). Qualitative data were described using number and percent. The Kolmogorov–Smirnov test was used to verify the normality of distribution Quantitative data were described using range (minimum and maximum), mean, standard deviation, median and interquartile range (IQR). Significance of the obtained results was judged at the 5% level. The applied tests include: Chi-square test (for categorical variables, to compare between different groups), Student t-test (for normally distributed quantitative variables, to compare between two studied groups), Mann Whitney test (for abnormally distributed quantitative variables, to compare between two studied groups), Wilcoxon signed ranks test (for abnormally distributed quantitative variables, to compare between two periods, and Spearman coefficient (to correlate between two distributed abnormally quantitative variables).

## Results

### Resiquimod Inhibits the mTOR-HIF-1α Axis in Both Malignant and Normal B Cells

**Fig. 1:**
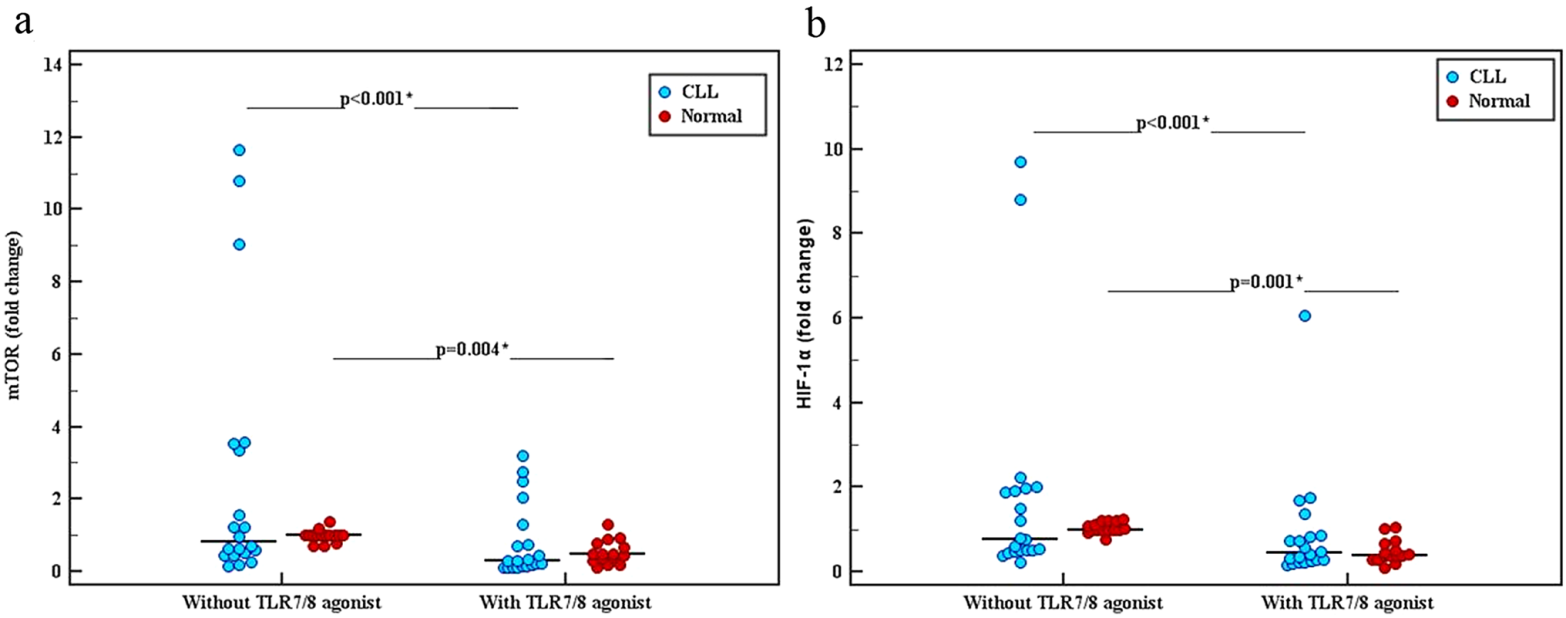
**a** Dot plot showing mTOR gene expression (fold change) in peripheral blood B cell with and without TLR7/8 agonist treatment in CLL (n = 20, *p* < 0.001) and Normal group (n = 15, *p* = 0.004). **b** HIF-1α gene expression (fold change) with and without TLR7/8 agonist treatment in CLL (n = 20, *p* < 0.001) and Normal group (n = 15, *p* = 0.001). Expression levels of each gene were normalized to GAPDH expression levels and adjusted to the levels in naïve B cells from healthy individuals (served as 1). Data shown are compared using median (IQR) values between CLL and normal group

We analyzed the expression levels of mTOR and HIF-1α in both isolated normal and malignant B cells after culturing for 48 h in presence and absence of TLR7/8 agonist. Regarding mTOR gene expression (Fig. 1a), mTOR expression median (IQR) values were 0.83 (0.47–3.45) in CLL group and 1.0 (1.0–1.01) in Normal group in untreated samples as shown in (Table 3). No statistically significant difference was found between both groups (*p* = 0.681). In treated samples (with TLR7/8 agonist), mTOR expression median (IQR) values were 0.31 (0.16–1.04) in CLL group and 0.49 (0.34–0.73) in Normal group with o statistically significant difference (*p* = 0.479). However, intra-group analysis revealed that median (IQR) values of mTOR gene expression were significantly lower in both CLL and Normal groups (*p* < 0.001, = 0.004) (Fig. 1a).

**Table 3:** mTOR and HIF-1α gene expression (fold change) in peripheral blood B cells with and without in vitro Resiquimod treatment in CLL and Normal groups

	CLL (n = 20)	Normal (n = 15)	*P*
mTOR			
Without TLR7/8 agonist			
Min.–Max	0.16–11.66	0.70–1.37	0.681
Median (IQR)	0.83 (0.47–3.45)	1.0 (1.0–1.01)	
With TLR7/8 agonist			
Min.–Max	0.12–3.20	0.12–1.30	0.479
Median (IQR)	0.31 (0.16–1.04)	0.49 (0.34–0.73)	
P_0_	< 0.001^*^	0.004^*^	
HIF-1α			
Without TLR7/8 agonist			
Min.–Max	0.23–9.70	0.77—1.25	
Median (IQR)	0.78 (0.50–1.95)	1.0 (1.0–1.16)	0.610
With TLR7/8 agonist			
Min.–Max	0.17–6.06	0.10–1.04	
Median (IQR)	0.44 (0.28–0.84)	0.39 (0.33–0.59)	0.681
P_0_	< 0.001^*^	0.001^*^	

IQR: Inter quartile range

P_0_: *p* value for Wilcoxon signed ranks test for comparing between with/without TLR7/8 agonist treatment in each of CLL and Normal groups separately

*P*: *P* value for Mann Whitney test for comparing between CLL and Normal groups

*: Statistically significant at *p* ≤ 0.05

As for HIF-1α gene expression (Fig. 1b), gene expression median (IQR) values were 0.78 (0.50–1.95) in CLL group and 1.0 (1.0–1.16) in Normal group in untreated samples as shown in (Table 3). No statistically significant difference in the expression of HIF-1α was found between both groups (*p* = 0.610). In treated samples (with TLR7/8 agonist), HIF-1α expression median (IQR) values were 0.44 (0.28–0.84) in CLL group and 0.39 (0.33–0.59) in Normal group. No statistically significant difference was observed between both groups (*p* = 0.681). However, intra-group analysis revealed that median (IQR) values of HIF-1α gene expression were significantly lower in both CLL and Normal groups (*p* < 0.001) (Fig. 1b).

### No Difference was Found in mTOR and HIF-1α Gene Expression Between Different Disease Risk Groups According to Rai staging

**Fig. 2:**
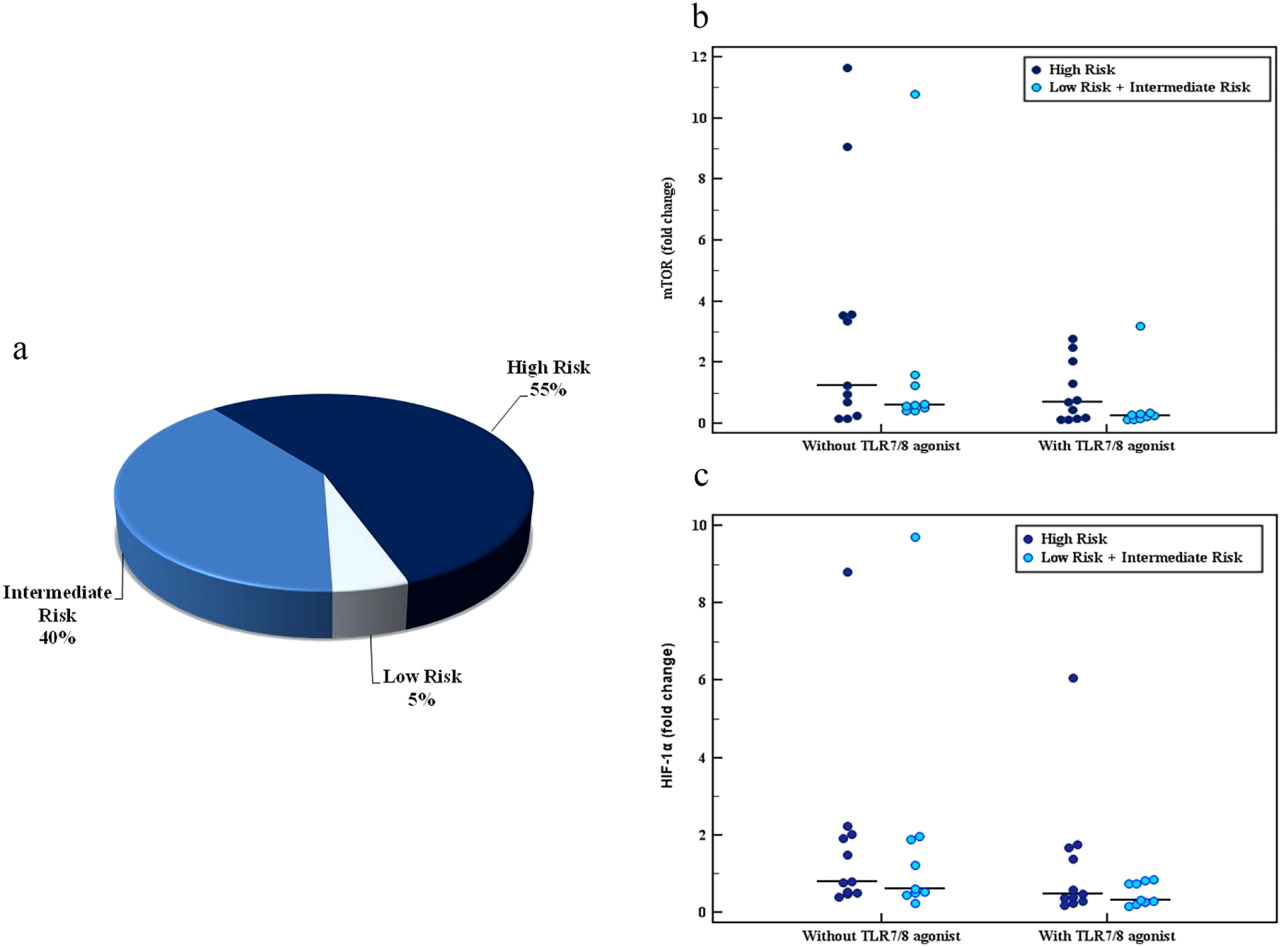
**a** Pie chart showing distribution of CLL patients (n = 20) according to Rai Staging system. **b** Dot plot showing mTOR gene expression (fold change) among different disease-risk CLL patients (n = 20) with and without TLR7/8 agonist addition. Although higher expression levels were seen among high disease-risk CLL patients, no significant difference was found (*p* = 0.331; 0.456). **c** HIF-1α gene expression (fold change) among different disease-risk CLL patients in the absence and presence of TLR7/8 agonist. No significant difference was found between groups either without or with TLR7/8 agonist (*p* = 0.603; 0.331, respectively)

We next compared the expression levels of both genes (mTOR and HIF-1α) in malignant B cells isolated from CLL patients with different disease-risk groups according to Rai staging system (Table 4). CLL patients (n = 20) were classified into three groups according to Rai staging system as shown in (Fig. 2a). Although high-risk disease patients had relatively higher expression levels of both genes when compared to (low + intermediate-) disease risk groups, no statistically significant difference was calculated whether in the presence or absence of TLR7/8 agonist (mTOR: *p* = 0.331; 0.456, Fig. 2b) (HIF-1α: *p* = 0.603; 0.331, Fig. 2c).

**Table 4:** Rai Staging of CLL patients (n = 20) in relation to mTOR and HIF-1α gene expression in B cells with and without TLR7/8 agonist treatment

	Rai staging		*P*
	High (n = 11)	Low + Intermediate (n = 9)	
mTOR			
Without TLR7/8 agonist			
Min.–Max	0.16–11.66	0.43–10.80	0.456
Median	1.24	0.62	
With TLR7/8 agonist			
Min.–Max	0.12–2.77	0.12–3.20	0.331
Median	0.71	0.24	
HIF-1α			
Without TLR7/8 agonist			
Min.–Max	0.39–8.81	0.23–9.70	0.610
Median	0.80	0.61	
With TLR7/8 agonist			
Min.–Max	0.18–6.06	0.17–0.85	0.331
Median	0.48	0.33	

*P*: *P* value for comparing between High and Low + Intermediate

## Discussion

Although treatable, CLL remains incurable with most patients relapsing or developing resistance to first-line treatment. Similar to most tumor cells, malignant CLL cells possess a distinct metabolic profile promoting their survival, chemo-resistance and immunoevasion mechanisms [22]. This metabolic reprogramming which follows aberrant activation of several metabolic checkpoint regulators, including mTOR and HIF-1α, represents a possible target for novel immunotherapeutic agents [23].

Eminent research evidence points to the involvement of TLR signaling in the regulation of immunometabolism in malignant cells and related microenvironment [8]. It has been proven that TLR8 signaling activation inhibits the immunosuppressive effect of melanoma and breast cancer-derived Treg cells. This metabolic rewiring was proven to be mediated via inhibition of glycolysis, used to fuel these immune-suppressive cells, by molecularly down-regulating mTOR-HIF-1α axis [24]. As for CD8^+^ T cell, stimulation of TLR7 signaling using its respective agonist (R848), was found to enhance the effector functions of murine and human CD8^+^ T cell in vitro. When investigating immunometabolic changes in TLR7-treated CD8^+^ T cells, up-regulation of glycolysis was found to be coordinated via Akt-mTOR-IRF4 pathway [25].

In the current study, we investigated the *in-vitro* effect of a TLR7/8 agonist, Resiquimod, on the gene expression of mTOR and HIF-1α as an indicator of metabolic reprogramming in malignant CLL B cells as compared to normal B cells. No significant difference was observed in mTOR and HIF-1α gene expression between CLL and normal groups either in absence or presence of Resiquimod. Similarly, no relation was observed between Rai staging of CLL patients and the gene expression of either mTOR or HIF-1α. However, intragroup analysis revealed that in vitro treatment of B cells with Resiquimod significantly decreased the expression of mTOR and HIF-1α genes in both CLL and normal groups.

Our findings go in accordance with previous studies reporting no significant alterations in HIF-1α mRNA expression levels in CLL patients compared to normal controls [20]. In addition, recent studies in CLL patients also reported insignificant associations between mTOR and HIF-1α expression levels and patients’ clinicopathological variables. However, a strong association was found between genetic aberrations such as Tumor protein 53 (TP53)-^dis^ and mutated-immunoglobulin heavy variable region (M-IGHV), which weren’t assigned in our study, and expression levels of mTOR and HIF-1α in CLL patients [20, 26, 27].

Accumulating data have proven that unlike normal B cells, CLL cells undergo metabolic alteration to support their pathological proliferation and chemo-resistance [22]. Among these metabolic alterations, several investigators have explored the functional aerobic glycolysis exhibited by CLL cells. For example, Ryland and his team proved that targeting *Warburg effect* in CLL cells was capable of inducing cell death *in-vitro* by down-regulation of glyceraldehyde 3-phosphate dehydrogenase (GAPDH), an enzyme involved in glycolysis pathway [28]. Another team explored metabolic plasticity of CLL cells using a panel of metabolic inhibitors. These Metabolic inhibitors, including glycolysis inhibitor (2-deoxyglucose) and oxidative phosphorylation (OxPhos) inhibitor (oligomycin), also resulted in cytotoxicity of CLL cells *in-vitro* [29]. These observations opened the door for the development of novel immune-therapeutics specifically targeting metabolic rewiring of malignant B cells.

In order to target the operational hyper-glycolysis observed in hematological malignancies, key metabolic regulators have been identified. Central to these metabolic regulators is the (mTOR-HIF-1α) pathway [30]. mTOR signaling is central in a variety of fundamental cellular processes including regulating cell growth, cell cycle control, autophagy and metabolism [31]. The latter involves multiple metabolic pathways such as glucose, lipid, amino acid and nucleotides metabolism. Thus, aberrant activation of mTOR signaling has been documented in a number of tumors including hematologic malignancies [30]. Furthermore, mTOR enhances the translation of HIF1α, the master oxygen sensing molecule, which fosters multiple tumor-promoting mechanisms such as angiogenesis, metastasis, cell proliferation and glucose metabolism [32]. The action of mTOR on cellular glycolysis includes induction of glucose transporters (GLUTs) such as GLUT-1 and other glycolytic enzymes promoting both cellular glucose uptake and glycolytic flux of cancer cells [33]. For instance, Frolova et al. showed that mTOR blockade, using Everolimus, diminished the glycolytic rate in acute lymphocytic leukemia (ALL) cells. This effect was mediated by downregulating HIF-1α expression which resulted in restoration of chemo-sensitivity of ALL cells [34]. Another study in acute myeloid leukemia (AML) cell lines showed that targeted inhibition of mTOR led to sensitization of AML cells to Aurora kinase inhibitors. Underlying mechanism was shown to involve glycolysis suppression leading to enhanced autophagy [35].

As for CLL, Hayun and his team were among the first researchers to examine the effect of mTOR inhibitor, Rapamycin, on CLL cells. They reported that CLL cells underwent apoptosis after treatment with Rapamycin through up-regulation of pro-apoptotic proteins and activating caspases machinery [36]. This was reassured by Marignac et al. through investigating the metabolic pattern exhibited by Desatinib-resistant CLL samples. Enhanced expression of glucose transporters was found to be associated with Desatinib resistant cells, which was targeted using inhibitors of mTORC1 or its downstream signaling pathways, leading to enhanced sensitization of these cells [37]. Consistent with previous study, Sharma and his team found that Fludarabine-resistant CLL cells experienced higher rates of glycolysis and OxPhos. Upon treatment with mTOR inhibitors, Rapamycin and Everolimus, cell death and sensitivity to Fludarabine was achieved in these cells [38]. Another study regarding Fludarabine resistant CLL cells found that HIF-1α was overexpressed, specifically in TP53- disrupted cells. Thus, its inhibition, using BAY87-2243, exerted a potent anti-tumor function within these cells overcoming Fludarabine resistance [27]. Furthermore, phosphatidylinositol-3-kinase (PI3K)/mTOR dual inhibiton in primary CLL cells was found to induce caspase-dependent apoptosis thus representing a powerful approach for treatment of CLL [39]. Finally, a recent study performed by Lu et al. analyzed proliferative drivers associated with CLL disease outcome. Their research proved that upregulation of mTOR signaling was linked to higher CLL- proliferative drive which is indicative of shorter lymphocyte doubling time, accumulation of genetic mutations and worse disease outcome in respective patients (Lu et al., 2021).

Preliminary studies on TLRs agonists in CLL mainly focused on TLR7 and TLR9, which showed heterogenous results. Early efforts studying the effect of TLR9 agonist, Cytosine-phosphorothioate-guanine oligodeoxynucleotides (CpG ODN), showed that treated samples experienced enhanced apoptosis. Apoptosis of treated CLL cells was proven to be caspase-dependent in addition to up-regulation of death receptors, such as Fas ligands [40]. Another group found an up-regulation of co-stimulatory molecules, CD80, CD86, pro-inflammatory cytokines production, especially TNFα, and sensitization to cytotoxic drugs in response to stimulation with TLR7 agonist using imidazoquinolines [41]. These findings were hypothesized to participate in the microenvironmental alteration of malignant CLL cells by enhancing the activity of tumor-reactive T cells as well as natural killer cells [42]. Subsequent deeper investigations on CLL subsets revealed that TLR7 and TLR9 stimulation results in heterogenous response according to IGHV mutation status. Mutated (M)-CLL cells were found to be more sensitive towards apoptosis after treatment with TLR ligands in contrast to unmutated (UM)-CLL [43]. However, subsequent studies uncovered paradoxical detrimental results of TLR1/2/6 resulted in survival of CLL leukemic cells through nuclear factor kappa-B (NF-κB) signaling [44]. In a similar manner, treatment of CLL cells with TLR7 and TLR9 agonists promoted their clonal expansion [45]. Lastly, indirect TLR antagonist interfering with TLR signaling pathways has been developed and tested in CLL. Recent inhibition of IL-1 receptor associated kinases-4 (IRAK4), an important signaling protein involved along TLR activation pathway, showed promising results by preferentially killing CLL cells [3].

Previous studies reported that B lymphocytes were activated upon culturing with TLR7/8 agonist in regard to cytokine and chemokine expression as well as the expression of co-stimulatory molecules and immunoglobulin production [46, 47]. By dissecting the metabolic profile of activated B cell population, an increase in glucose uptake following B cell stimulation has been reported by several studies. Doughty et al. found that upon BCR crosslinking, B lymphocytes rapidly increase their glucose uptake and subsequent glycolysis. BCR-mediated enhanced glycolysis was found to involve PI3K pathway as evidenced by the inhibition of glycolysis upon PI3K deficiency [48]. Cho S. and his team reassured previous findings by proving that survival and proliferation of B cells were dependent upon glucose metabolism [49]. A later study investigated the effect of glycolytic inhibition, using a pyruvate dehydrogenase (PDH) kinase inhibitor, on the activation and proliferation of B cells. Their findings showed a sharp suppression in B cells proliferation and antibody secretion in vivo and *in-vitro.* Furthermore, deletion of Glut-1 in B cells led to reduction in numbers as well as impairment in functions [50]. These data led to assuming that glycolysis is increased, by default, to support B cell activation. However, a subsequent study proved that although the uptake of glucose is increased, glycolytic metabolites along with lactate levels were found to decrease upon activation. These results suggested that glucose is being routed into alternative pathways in activated B cells. Further analysis showed that OxPhos and Tri-carboxylic acid (TCA) cycle pathways were up-regulated signifying their key roles in stimulated B cells [51]. This latter finding goes in accordance with our results showing a decrease in glycolytic metabolic mediators, mTOR and HIF-1α, upon stimulation of naïve B cells with TLR7/8 agonist.

As mentioned before, our study lacked genetic mutations’ investigations which is recommended to be collected and correlated with metabolic regulators on a larger sample size in future studies. Also, further metabolomic investigations to assure the observed effect of TLR7/8 agonist on immunometabolism of B cells such as measuring glucose levels or lactate levels are recommended. Moreover, further studies on the effect of TLR7/8 agonist on the metabolism of other types of leukemia will be of great value. In summary, we present a novel finding regarding the effect of Resiquimod on leukemic CLL cells’ metabolism. By employing genetic sequencing using qRT-PCR, we uncovered a significant decrease in genes supporting hyper-glycolytic capacity of malignant B cells when targeting them with TLR7/8 agonist. Accordingly, targeting metabolic pathways in leukemic B cells by TLR agonist could be used as a possible novel immunotherapeutic adjuvant.

### Supplementary Information

Below is the link to the electronic supplementary material.

Supplementary file1 (PDF 99 kb)
