# Supplementary material for: In-vitro Modulation of mTOR-HIF-1α Axis by TLR7/8 Agonist (Resiquimod) in B-Chronic Lymphocytic Leukemia
Source: Indian J Hematol Blood Transfus. 2023 Apr 7;39(4):537–45. doi: 10.1007/s12288-023-01649-y (PMC10542076; doi:10.1007/s12288-023-01649-y)
Supplement: Supplementary file 1 — Supplementary file1 (PDF 99 kb) [file 12288_2023_1649_MOESM1_ESM.pdf]

APPENDIX I

Research Ethics Committee Standard Operating Procedures

Italic text in brackets [ ] indicate instructions to the reader.

A. Introduction

The *[state name of Faculty or Institution]* is committed to high-quality research on all aspects of the health and behavior of people, and such research is possible only through the participation of humans as subjects in research.

While the primary goal of research is to enhance the well-being of society, an important objective of research involving human subjects is protection of the rights and welfare of subjects who participate in research. Accordingly, research should be guided by the ethical principles embraced by the Declaration of Helsinki and *[state other document(s) if relevant]*. These principles include autonomy (respect for persons), beneficence (protecting subject welfare), non-maleficence (minimizing potential harms of research), and justice (avoidance of exploitation). Justice also requires that the benefits and burdens of research be distributed fairly among all groups and classes in a society, as well as between the different countries who are participating in the research.

B. Assurances

The *[state Dean or President or other high-ranking official]* will oversee the research practices in the *[state Faculty or Institute]* and assures that these practices will conform to the principles of research ethics. Part of this assurance includes the establishment of an appropriately constituted Research Ethics Committee (REC), which shall have the responsibility to review and monitor research involving human subjects.

C. REC Mission and Authority

1. Scope and Purpose

The purpose of the REC is to protect the rights, safety, and welfare of all research subjects. To achieve this, the REC must advise investigators in designing research projects in a manner to minimize potential harm to human subjects, review all planned research involving human subjects prior to initiation of the research, approve research that meets established criteria for protection of human subjects, and monitor approved research to ascertain that human subjects are indeed protected.

2. REC Responsibility and Authority

All human subjects research carried out at *[state name of Faculty or Institution]* must be reviewed and approved or determined exempt by the REC prior to the involvement of human subjects in research.

Accordingly, the REC has the following responsibilities and authority:

- The REC shall review and have authority to approve, require modifications in (to secure approval), or disapprove initial and continuing reviews of all research activities.
- The REC shall have authority to suspend or terminate approval of research that is not being conducted in accordance with the REC's requirements or that has been associated with unexpected serious harm to subjects.
- The REC must report to the *[Dean or President]* unanticipated problems involving risks to subjects and others or serious or continuing noncompliance by investigators.

D. Constitution of the REC

The REC will be constituted to ensure a) competent review of the ethical aspects of the research and b) independence from influences that could affect the performance of unbiased reviews.

1. Chairperson

a. Appointment: The chairperson will be appointed directly by the *[Dean or President or other high-ranking official of the Faculty or the Institute, neither of which should be involved in the committee to ensure independence of the REC from institutional influence]*.

[*Alternatively, to ensure more independence, the chairperson could be elected by the Faculty Council or by an initial core group of a committee (members with experience in research ethics) who were appointed by a high-ranking institutional official.]*

b. Qualifications of the chair: The chairperson shall have the following qualifications:

i. A professor on the academic staff (if in a university)
ii. Reasonable experience in performing research
iii. Basic training in research ethics
iv. Reasonable communication skills and leadership characteristics
v. Committed to the protection of human subjects in research

c. Term of appointment: The chairperson shall serve for a period of three years. Afterwards, the appointment of the chairperson could be renewed by reappointment by the *[Dean of the Faculty or the President of the Institution]*. The chairperson shall not serve for more than two consecutive three-year terms. [*To ensure more independence, the chairperson's appointment could be renewed by a Faculty Council.]*

d. Responsibilities: The chairperson shall be responsible for the actions of the REC, including the scheduling of regular meetings and communications between the REC, members of the research staff, and institutional officials. It is expected that the chairperson will preside over more than three-quarters of the convened meetings of the REC.

e. Vice-Chairperson: The chairperson will choose a vice-chairperson to help him or her in carrying out his or her responsibilities. The vice-chair will carry out the chairperson duties in his/her absence upon written permission from the chairperson.

2. Members of the RECs

a. Members: Members of the RECs will reflect a multidisciplinary and multisectorial composition, including relevant scientific expertise (i.e., appropriate to the types of protocols that will be reviewed), balanced age and gender distribution, a mix of junior and senior staff members, and a mix of medical, scientific, and nonscientific persons including nonaffiliated lay representatives (e.g., lawyer, journalist) to reflect the different viewpoints of the community.

b. Numbers: The number of persons in the REC should be kept fairly small, between seven and 11 members. It is generally accepted that a minimum of five persons is required to compose a quorum. There is no specific recommendation for a widely acceptable maximum number of persons, but it should be kept in mind that too large a REC will make it difficult in reaching consensus. Hence, 12-15 is the maximum recommended number.

c. Qualifications: Members will include the following:

i. holding at least a college degree
ii. the nonaffiliated community representative is exempted from having a college degree to ensure proper representation of a large sector of the community who might not have such qualification
iii. have an interest in research issues and research ethics
iv. be reputable and trustworthy
v. be willing to volunteer their time and effort
vi. be willing to sign a confidentiality agreement regarding meeting deliberations, applications, information on research subjects, and other related matters

d. Conditions of Appointment: Each member shall:

i. agree to meet all education and training requirements
ii. sign a confidentiality agreement regarding meeting deliberations, applications, and information on research subjects

e. Appointment Process

i. Initial constitution of the REC: An initial core group of members shall be selected directly by *[Dean, President, or Faculty Council]*. This core committee will identify, interview, and then choose by consensus the

subsequent members of the committee.

ii. Appointment of subsequent members: The REC will identify prospective members and review with them the nature and demands of serving on the REC. If the member is willing to serve, then the chair and vice-chair shall seek approval from a governing body of the department or faculty (e.g., faculty council). Upon approval, the full REC will, by consensus, approve the selection of the prospective member.

iii. Conflicts of interest should be avoided when appointments are made, but if unavoidable, there should be transparency and management of the conflict of interest with regard to such interests on a case-by-case basis.

f. Terms of Appointment

i. Duration: Each member shall be appointed for a cycle of three years in duration.

ii. Renewal: At the end of each cycle of appointment, members wishing to stay on should make a written request to the chairperson. Subsequent renewal will depend on prior quality of work and attendance performance and be determined by a consensus of the full committee.

iii. Resignation: Members wishing to terminate their appointment prior to the three-year cycle shall send a written letter of resignation to the chairperson two months in advance in order to have enough time to appoint a another member.

iv. Disqualification: Members may be asked to leave the REC by a written letter from the chairperson if any of the following occurs:

1) Failure to attend three consecutive meetings without permission or more than half of the meetings per year
2) Negligence in reviewing protocols
3) Breach of confidentiality agreement
4) Termination shall be decided by a majority vote of the full REC.

g. Orientation and Training of IRB Members:

Initial Education: Following appointment the new member will go through the REC orientation, which consists of an introductory lecture followed by an informational session on practical matters with the REC chair. Subsequent education may take one of the following types:

i. Previously held workshop (of at least two days duration) in research ethics

ii. Successful completion of a Web site training course in research ethics.

Continuing education: An REC should set standards for continuing education of its members every three years (e.g., regularly scheduled review of published articles in research ethics, attendance at workshops, lectures, seminars, etc.).

h. Conflicts of Interest: No IRB may have a member participate in the IRB's initial or continuing review of any project in which the member has a conflicting interest, except to provide information requested by the IRB.

Examples of such conflicts of interest could include: a member of the IRB who serves as an investigator on research under consideration by that IRB; or a member who holds a significant financial interest in a sponsor or product under study.

3. Independent Consultants

The REC may, at the discretion of the chair or its members, invite individuals with competence in special areas to assist in the review of issues that require expertise beyond or in addition to that available on the REC. These individuals may not vote with the REC. Consultants are not included in determining or establishing a quorum at the meetings. REC meeting minutes reflect the presence of consultants.

E. REC Research Review Evaluations Procedures, Criteria, and Actions

The REC is charged with the responsibility for reviewing and monitoring human subject research conducted under the mandate of *[name of Faculty or Institution]*. Therefore, the first question with respect to REC review of a project is a determination of whether the project fits the definition of research.

a. Is It Research? Research is defined as "a systematic investigation, including development, testing, and evaluation, designed to develop or contribute to generalizable knowledge." Thus, a key aspect of research is that there should be a systematic design in advance, generally utilizing a scientific approach or protocol, for the defined purpose of contributing to generalizable knowledge. Research can encompass a wide variety of activities, which includes experiments, observational studies, surveys, tests, and recordings.

b. Does It Involve Human Subjects? A human subject is defined as "a living individual about whom an investigator conducting research obtains (1) data through intervention or interaction with the individual, or (2) identifiable private information."

Identifiable private information "includes information about behavior that occurs in a context in which an individual can reasonably expect that no observation is taking place" (such as a public restroom) "and information which has been provided for specific purposes by an individual and which the individual can reasonably expect will not be made public (for example, a healthcare record)."

Intervention includes physical procedures, manipulations of the subject, or manipulations of the subject's environment for research purposes.

Interaction includes communication between the investigator and the subject. This includes face-to-face, mail, and

phone interaction as well as any other mode of communication.

1. Meeting Frequency

The REC will meet at regular time intervals in accordance with the needs of the workloads. Generally the REC should meet at least once a month on a regularly scheduled day (for example, every two weeks, every month, etc.). In certain circumstances, RECs can meet on an "as needed" basis.

Scheduled meetings may be cancelled by the chair due to a) an insufficient number of applications requiring review at a convened meeting, b) inability to secure a quorum for attendance, or c) other reasons as may arise that make a scheduled meeting unnecessary or otherwise inappropriate.

2. Quorum Requirements

a. The number required to compose a meeting will be half of the members with a minimum of five.

b. No quorum will consist entirely of members of one profession (e.g., medicine) or gender.

c. A quorum will include at least one member who is non-affiliated with the institution.

3. Submission of Applications for New Studies

a. Persons Submitting: The principal investigator should submit an application for review of the ethics of a proposed research project.

b. Materials Submitted: Each application should consist of the following:

- A signed and dated application form (developed by the REC)
- Full protocol
- Consent form
- Product brochure for new drug/device
- Time plan for the study
- CVs for the principal and co-investigators
- Copies of actual questionnaires to be used in the study
- Copies of materials to be used (e.g., advertisements) for the recruitment of potential research subjects.
- Signed investigator assurance agreement to comply with ethical principles and legal requirements set out in relevant laws and guidelines.

If the application is incomplete or otherwise not fully prepared for review, the REC shall return it to the investigator with a request for necessary changes and additional information.

c. Deadlines:

i. Submission: The deadline for submission will be at least 15 days prior to the date of the meeting review.

ii. Investigator notification: Investigators will be notified of an REC decision within 48 hours after a decision has been reached.

4. Review of Applications of New Studies

[An REC may elect to use a primary reviewer system in which one or more members are assigned to lead the review and present the protocol for discussion at the convened meeting. Alternatively, all REC members are provided with detailed materials describing the research so that each member will be able to discuss the protocol at the meeting.]

a. Member Review:

1. A member will be selected to be the primary reviewer of the protocol and will be responsible for:
   a. Completing the primary reviewer form
   b. Presenting the protocol for discussion at the meeting
2. All members shall receive protocols for review at least one week prior to the review meeting
3. All members are required to review all submitted materials and be prepared to discuss all protocols at the convened meeting.

b. REC Evaluation Criteria: The REC will assess the following review criteria:

- Acceptable Social Value to the community/country.
- Scientific Design: The REC will consider the assessment of scientific design as determined by a separate Research Committee. The REC will consider elements of scientific design, related to ethical issues, not reviewed by the Research Committee (e.g., justification of the use of placebo control arms, inclusion and exclusion criteria, etc.).
- Recruitment of Research Subjects: In accordance with Belmont principles, both the burdens and benefits of research should be distributed equitably. Selection of subjects is one important means of ensuring that the burdens and benefits of research are distributed equitably. In making this assessment the REC will take into account the purposes of the research and the setting in which the research will be conducted, and should be particularly cognizant of the special problems of research involving vulnerable populations, such as children, prisoners, pregnant women, mentally disabled persons, or economically or educationally disadvantaged persons. If such vulnerable populations will be potentially enrolled in research, then the REC will determine the appropriateness of additional safeguards to provide added protection to vulnerable populations.
- Analysis of Risks and Benefits: The REC will identify all risks (physical, psychological, social, and economic) involved in the research. Risks to subjects must be minimized by using procedures that are consistent with sound research design and that do not unnecessar-

ily expose subjects to risk, and whenever appropriate, by relying on procedures already being performed on the subjects for diagnostic or treatment purposes. Risks to subjects must be reasonable in relation to anticipated direct benefits, if any, to subjects, and the importance of the knowledge that may reasonably be expected to result from the research project. Each protocol will be assigned a risk level (minimal risk, greater than minimal risk, or too risky (in the last case, the protocol will be disapproved).

- Privacy of Subjects and Confidentiality Procedures to Protect Subjects' Data: The REC will determine the appropriateness of procedures in place to ensure subject privacy and to ensure the confidentiality of data obtained from the subjects.
- Procedures to Monitor Subjects During the Study: The REC will consider the appropriateness of criteria for prematurely withdrawing research subjects for safety considerations (if applicable); the adequacy of provisions to monitor safety of research subjects; and the determination of whether a Data Safety Monitoring Board (DSMB) is required.
- Informed Consent: Unless specifically waived by the REC, informed consent must be sought from each prospective subject or the subject's legally authorized representative. The REC shall also:
  - Review the adequacy, completeness, and understandability of written and oral information.
  - Determine whether signed, written informed consent can be waived and the validity of alternative procedures to document the provision of informed consent (e.g., thumbprint or verbal, witnessed consent).
  - Determine whether informed consent could be obtained from the subject's legally acceptable representative.
  - Determine whether the informed consent document contains the required basic elements of consent (see checklist).
- Externally Sponsored Studies: Sometimes research is undertaken in Egypt, but sponsored, financed, and sometimes entirely or partly carried out by an external international or national organization or pharmaceutical company in collaboration with or with the agreement of the appropriate authorities, institutions, and personnel of Egypt. In such externally sponsored research, the REC in *[state the name of the Faculty or Institution]* and in the country of the sponsor shall have responsibility for conducting both scientific and ethical review, as well as the authority to withhold approval of research proposals that fail to meet their scientific or ethical standards.

  The REC at *[state the name of Faculty or Institution]* shall have the following special responsibilities:
  - Determine whether the objectives of the research are responsive to the health needs and priorities of Egypt to avoid exploitation of underprivileged communities.
  - Obtain information regarding the type of post-trial benefits to the community and Egypt to determine that the burdens and potential benefits of the research have been fairly distributed between the participating countries.
  - Determine whether the research plan conflicts with the involved community's customs and traditions.

c. Expedited Review:

- Certain minimal risk protocols may receive expedited review by the chairperson. All expedited decisions shall be communicated to the next convened meeting of the REC. The REC shall establish criteria by which protocols can be reviewed by such an expedited procedure.
- "Minimal risk" means that the probability and magnitude of harm or discomfort anticipated in the research are not greater in and of themselves than those ordinarily encountered in daily life or during the performance of routine physical or psychological examinations or tests.

5. Voting and Decision Making

a. Participation: All members who attended the meeting and discussed the protocol will participate in the voting unless a member has a conflict of interest. Those members physically present for the vote should be recorded as either voting for, against, or abstaining, without identification by names. Members who are excused from the vote (e.g., due to conflict of interest) should physically leave the room, would not be counted in the aforementioned tally, and should be identified by name in the minutes.

b. Quorum: Decisions should be made at meetings where a quorum is present.

c. Consensus: Decisions should be arrived at through consensus, where possible. In cases where a consensus appears unlikely or when discussions become prolonged, the chairperson shall call for a vote. In such instances, a majority vote will be sufficient to arrive at a decision. In case of a tie, the decision favored by the chairperson shall be determinate.

d. Conflict of Interest: When an REC member has a conflict of interest (see D.2(h):Member conflict of interest) that requires him/her to excuse himself/herself from discussion of and voting on a particular protocol, that member should leave the meeting room for the duration of the discussion and vote, except as requested to address questions raised by other members. If the member's conflict of interest causes a loss of

quorum, the vote should be postponed to another meeting. For this reason, REC members should notify the chair prior to the meeting if they have a conflict of interest related to a specific protocol slated for review at the meeting, and every effort should be made to ensure transparency by full disclosure of such conflict of interest.

e. Types of Decisions allowed:

- ***Approval:*** Approval of research. In the case of an approval with no changes, the research may proceed once the PI receives written documentation of REC approval.
- ***Approval with minor changes:*** The REC may determine that a study may be approved with stipulated minor changes or clarifications. Minor changes are those changes that do not involve potential for increased risk or decreased benefit to the human subjects. Some examples of minor changes are: changes in contact information or identity of non-key research personnel, changes in the study title, and changes in the consent form that reflect the minor changes listed earlier.

  For minor changes, the chair or a voting REC member(s) designated by the chair must ensure that the investigator makes the appropriate changes to the research protocol. Such changes must be clearly delineated at the convened meeting so that subsequent review requires simple verification of concurrence. The research may proceed after the required changes are verified and the designated reviewer approves the protocol.
- ***Deferral:*** The term "deferral" is used to describe the situation in which the REC determines that substantive changes must be made before approval may be granted. The investigator's response, including any amended materials, must be reviewed by the convened REC.
- ***Disapproved:*** The project, as proposed, is disapproved and may not go forward. Disapproval usually indicates that a proposal requires major changes not likely to be feasible without a complete reassessment of the protocol by the investigator and/or sponsor.
- ***Suspension and termination of research study by REC:*** The chair of the REC or the convened REC may suspend a study at any time if it is determined that the study requires further review or evaluation. This determination may be made due to an adverse event, noncompliance, or other danger to human subjects. Once a study is suspended, the convened REC should review the study and either require changes to the protocol, allow the study to restart, or terminate the study. Though the chair may suspend a study, only the convened IRB can make the decision to terminate a study.

f. Appeal of REC Decisions: Investigators may appeal the REC's decisions. At the discretion of the chair, the investigator may make such an appeal in writing to the REC. At the REC's discretion, the investigator may be invited to the REC meeting at which his or her appeal will be considered.

g. The follow-up intervals will be determined according to the level of risk of the protocol. In general, duration of approval will be a maximum of one year.

h. REC Meeting Minutes should be in sufficient detail to show the following:

Attendance at the meeting:

- date and time meeting starts and ends
- names of members present
- names of members absent
- names of alternates attending in lieu of specified absent members
- names of consultants present
- names of investigators present
- names of guests present

Actions taken by the REC:

- Actions taken by the REC at a convened meeting as well as the vote on these actions including the number of members voting for, against, and abstaining, and (if applicable) notation that any members with a conflict of interest (identified by name) were excused and were absent for the discussion and vote;
- The basis for requiring changes in or disapproving research (see 7.4 below);
- For each protocol in which changes are stipulated by the REC, a determination of whether the changes represent minor modifications that do not require verification by the convened IRB, or whether they are significant, requiring convened IRB review; and,
- A written summary of the discussion of controversial issues and their resolution.

REC findings and determinations: The following are required findings and determinations, and must be noted in the minutes with reference to the appropriate country regulations:

- Determination of the level of risk for human subjects in the research study (no citation required);
- Justification for waiver or alteration of informed consent;
- Justification for the waiver of the requirement for written documentation of consent;
- Justification for approval of research involving children and other vulnerable groups;
- Justification for approval of research planned for an emergency setting; and
- Special protections warranted in specific research projects for groups of subjects who are likely to be vulnerable to coercion or undue influence, such as children, prison-

ers, pregnant women, mentally disabled persons, or economically or educationally disadvantaged persons.

The secretary of the REC will be responsible for taking the minutes of the meeting. At each meeting, one member of the committee will take notes and review the minutes to ensure accuracy and completeness.

6. Communication of Decisions

a. A decision of the REC shall be communicated to the investigator in writing within three working days of the meeting.

b. Each decision shall include:

- A clear statement of the decision reached
- Justifications of any disapproval
- In cases of conditional approval, a list of the conditions needed for approval and its associated justifications
- In cases of a positive decision, a statement of the responsibilities of the investigator is issued (e.g., confirmation of the acceptance of any requirements imposed by the REC, submission of progress reports, the need to notify the REC in cases of protocol amendments, changes to recruitment materials, changes to the consent form, and the reporting of any unexpected adverse events or unanticipated problems or termination of the study)
- The date and place of the decision
- Any advice given by the REC
- Signature of the chairperson

7. Investigators' Responsibilities During Conduct of the Study

During the conduct of the study, the investigator shall submit within a specified period of time (to be determined for each category) the following:

- Amendments to the protocol
- Serious and unexpected adverse events
- Safety reports (if applicable)
- Reports of any Data and Safety Monitoring Board
- Unanticipated problems
- Termination of the study

The REC will determine which of the above can be reviewed by an expedited procedure and which requires full committee review.

8. Continuing Review

a. Submission: At the time of continuing review, the investigator shall submit the following information for review:

- Enrollment of subjects: gender and age
- Number of subjects withdrawn and reasons for such withdrawal
- Adverse events (cumulative and type for the previous period since the last review)
- Modifications to the protocol
- Changes of investigators
- Results, if available
- Current informed consent form

RECs should determine which continuing reviews can be reviewed by an expedited process and which continuing protocols require full committee review.

b. Lapsed Studies: A lapsed study is one for which the approval period has expired prior to the renewal of approval by the REC. If the investigator fails to submit the materials for continuing review prior to the REC meeting that needs to review the study before the expiration date, then the lapsed study will be classified as inactive. Once a study has lapsed, notification should be sent to the investigator ordering that all study-related measures must immediately cease except those necessary for welfare of the human subjects. If the investigator desires to continue a study that has lapsed for more than one month, then the investigator must submit a new application for re-review by the REC, and must wait for REC approval before resuming research under the protocol.

F. Waiver of Informed Consent

The REC may approve a consent procedure that does not include, or that alters, some or all of the elements of informed consent set forth above, or waive the requirement to obtain informed consent, provided the REC finds and documents that:

- The research involves no more than minimal risk to the subjects;
- The research could not practicably be carried out without the waiver or alteration.

Alternatively, the REC may waive the requirement for informed consent involving research in the emergency setting. *[RECs must develop criteria under which informed consent may be waived.]*

G. Short Form Consent Procedures

There may be circumstances when a subject is unable to read the full consent document (e.g., when the subject is illiterate or does not speak the language in which the consent document is written). In such cases, a short form may be used. A short form is a written consent document stating that the required elements of informed consent have been presented orally to the subject or the subject's legally authorized representative. When this method is used, there shall be a witness to the oral presentation (not a member of the research team). Also, the REC shall approve a written summary of what is to be said to the subject or the representative. Only the short form itself is to be signed by the subject or the representative. However, the witness shall sign both the short form and a copy of the summary, and the person actually obtaining consent shall sign a copy of the summary. A copy of the summary shall be given to the subject or the representative, in addition to a copy of the short form.
